# ^1^H NMR metabolomics insights into comparative diabesity in male and female zebrafish and the antidiabetic activity of DL-limonene

**DOI:** 10.1038/s41598-023-45608-z

**Published:** 2024-02-15

**Authors:** Khaled Benchoula, Christopher J. Serpell, Ahmed Mediani, Abdulaziz Albogami, Norazlan Mohmad Misnan, Nor Hadiani Ismail, Ishwar S. Parhar, Satoshi Ogawa, Wong Eng Hwa

**Affiliations:** 1 School of Medicine, Faculty of Health and Medical Sciences, Taylor’s University, 1, Jalan Taylors, 47500 Subang Jaya, Selangor Malaysia; 2 School of Pharmacy, University College London, London, WC1N 1AX UK; 3 Institute of Systems Biology (INBIOSIS), Universiti Kebangsaan Malaysia, 43600 UKM Bangi, Selangor Malaysia; 4 Biology Department, Faculty of Science, Al-Baha University, 65779-7738 Alaqiq, Saudi Arabia; 5 Institute for Medical Research Malaysia, No.1, Jalan Setia Murni U13/52, Seksyen U13, Setia Alam, 40170 Shah Alam, Selangor Darul Ehsan Malaysia; 6 Atta-ur-Rahman Institute for Natural Products Discovery, UiTM Puncak Alam Campus, 42300 Puncak Alam, Selangor Malaysia; 7 Monash University (Malaysia) BRIMS, Jeffrey Cheah School of Medicine and Health Sciences, Jalan Lagoon Selatan, Bandar Sunway, 47500 Subang Jaya, Selangor Malaysia

**Keywords:** Biochemistry, Drug discovery, Molecular biology

## Abstract

Zebrafish have been utilized for many years as a model animal for pharmacological studies on diabetes and obesity. High-fat diet (HFD), streptozotocin and alloxan injection, and glucose immersion have all been used to induce diabetes and obesity in zebrafish. Currently, studies commonly used both male and female zebrafish, which may influence the outcomes since male and female zebrafish are biologically different. This study was designed to investigate the difference between the metabolites of male and female diabetic zebrafish, using limonene – a natural product which has shown several promising results in vitro and in vivo in treating diabetes and obesity—and provide new insights into how endogenous metabolites change following limonene treatment. Using HFD-fed male and female zebrafish, we were able to develop an animal model of T2D and identify several endogenous metabolites that might be used as diagnostic biomarkers for diabetes. The endogenous metabolites in males and females were different, even though both genders had high blood glucose levels and a high BMI. Treatment with limonene prevented high blood glucose levels and improved in diabesity zebrafish by limonene, through reversal of the metabolic changes caused by HFD in both genders. In addition, limonene was able to reverse the elevated expression of AKT during HFD.

## Introduction

Zebrafish have been successfully used as disease models in pharmaceutical discovery for drug screening. The model reduces housing, maintenance, and food costs compare to other laboratory models such as rodent. Administration of a high-fat diet and/or overfeeding could affect the metabolism of zebrafish by changing the expression of glycolysis, lipogenesis, gluconeogenesis, and protein metabolism enzymes and hormones^1^. Diabetic induction agents such as STZ and alloxan, which have been used for decades to induce diabetes in different animal models, could also induce diabetes in zebrafish^2^. Most obesity and diabetes studies in zebrafish tend to use males due to the concern that oestrous cycles might cause data variability. However, this theory is based on research in other animal models, not zebrafish. Thus, the first major goal of this study is to investigate the difference between diabetic male and female zebrafish metabolites using a metabolomics approach.

Limonene is a naturally occurring monocyclic monoterpene found in a variety of plants and fruits, including citrus fruits, cherries, and mint. it’s under investigation as a promising medical agent used to treat assorted medical contentions including diabetes, and obesity^3^ The fundamental approach to addressing diabetes involves shifting to an alternate mechanism, yet the ultimate mechanism that is predominantly observed involves stimulating β-cells to release more insulin^4,5^. Santiago et al. confirmed that limonene can treat insulin resistance by reducing the mass of β-cells in the pancreas in a high-fat diet-induced diabetes animal model. Limonene has also been reported to prevent liver damage by reducing expression of certain hepatic enzymes including aspartate aminotransferase, alanine aminotransferase, and alkaline phosphatase which enhance the liver damage and insulin resistance^6^. However, more research into the underpinning pathways that limonene may activate is required. As a result, the second primary purpose of this work is to evaluate the altered pathway upon limonene treatment in diabetic male and female zebrafish models.

## Material and methods

### Zebrafish maintenance

Wild-type male and female adult zebrafish (*Danio rerio*) at 3 months of age were maintained in 9 L tanks at Taylor’s University. The fish was obtained from a local supplier (3B Aquatics, SDN BHD, Kuala Lumpur, Malaysia). The water was replaced daily, well-aerated, dechlorinated and kept at 28 °C, pH 6.8–7.5, and salinity of 800–1200 S/cm. The light/dark circle was dictated manually at 10 h /14 h. Zebrafish were fed twice daily using artemia adult zebrafish food which contains 22% fat, 44% proteins, and 16% carbohydrates. The experimental procedures were approved by the Commission of Ethics at Monash University, Malaysia. the research was conducted following the internationally accepted principles for laboratory animal use and care as found in the European Community guidelines (EEC Directive of 1986; 86/609/EEC).

### Induction of diabetes in adult zebrafish

Overfeeding with an HFD was assessed to induce type 2 diabetes-related obesity in adult zebrafish. The protocol followed those previously described^2,7^, with the following modifications. Wild-type male and female zebrafish groups (n = 8) were overfed for 4 weeks with an HFD containing egg yolk powder (59% fat, 32% proteins, 2% carbohydrates; 30 mg/fish for 3 times per day) and artemia (22% fat, 44% proteins, 16% carbohydrates; 5 mg/fish for twice daily). However, the control groups (n = 8) were fed once daily using artemia (5 mg/fish). The fish were maintained as 50 fish in 9 L tanks with changing the water twice daily, while pH and aeration were maintained manually. The BMI and fasting blood glucose levels were measured after 4 weeks of the diet program. The fish were not fed for 24 h before blood was taken. To achieve this, the fish was anaesthetized using cold water (0 °C), and then a needle of an insulin syringe was pushed gently under the gill to reach the heart and took a maximum of 10 µL of blood. The blood was inserted immediately into glucose strips of EasyTouch^®^ glucometer to measure the fasting blood glucose level^2^.

### Oral glucose tolerance test (OGTT)

The oral glucose tolerance test (OGTT) is a routine laboratory test in which a patient with diabetes is given a glucose solution to examine the capability of the body to process the glucose, β-cell function, and insulin resistance. A group of diabesity zebrafish with a control group (n = 18; 6 fish at each time point) was anaesthetized and was given a glucose solution orally at a dose of 1.25 mg/g body weight. The fish were allowed to recuperate before being anaesthetized again at 0, 30, and 120 min to collect blood and assess blood glucose levels^7^.

### DL-limonene anti-diabetes activity investigation

The established diabesity ebrafish model was used to test the effectiveness of DL-limonene. DL-Limonene (> 98%) was purchased from Sigma-Aldrich^®^ (Germany). Based on preliminary toxicity testing (not shown), we used two dosage levels of the compound: a low dose (5 mg/L; n = 20 males and 20 females), and a high dose (20 mL/L; n = 20 males and 20 females). Methanol (AR grade; Fisher Scientific^®^, UK) was used to dissolve limonene in this experiment since it has been documented that methanol is a safe solvent for zebrafish at concentrations ranging from 0.1 to 1%^8,9^. The practical solution was prepared by mixing (1:3; v/v) of DL-limonene in methanol and then mixed with 1 L of water to reach the stated desired concentration. Overfed high-fat diet zebrafish were used in this experiment. In addition to these two groups, an untreated overfed with HFD group (n = 20 male and 20 female), a SD (Standard Diet) group (n = 20 male and 20 female), a HFD group treated with methanol (60 µL/L; n = 20 male and 20 female) fed with HFD and treated group with methanol (60 µL/L; n = 20 male and 20 female) fed SD was used in this experiment.. The methanol solution group was prepared by using the greatest concentration of methanol utilized to make 20 mg/L of DL-limonene, which is 60 µL (1:3; v/v). Every day for 4 weeks, the treated group was submerged overnight (14 h) in a freshly prepared treatment solution of DL-limonene or methanol. The fasting blood glucose level was measured after overnight fasting using a glucometer. The fish's body weight and length were measured for BMI calculation before withdrawing the blood. The fish’s water was changed twice daily (morning and evening) and aeration was provided manually. The fish was sacrificed in ice water and was reserved in (-80) for the next analysis.

### ^1^H NMR fingerprinting

The sacrificed fish was submerged in liquid nitrogen to arrest the metabolism and store at − 80 °C for later use. 1 g of tissue was mixed and then homogenized with 4 ml of methanol and 0.85 mL of water, and then 2 mL/(g of tissue) of chloroform was added after vortexing of the sample. The mixture was vortexed again and a further 2 mL/g of chloroform and 2 mL/g of water were added before vortexing again. The mixture was stored in the fridge for 15 min prior to centrifugation for 15 min at 1000 × g at 4 °C. The solution was separated into two phases: polar metabolites (upper phase) and lipophilic metabolites (lower phase). The solvent was removed using a speed vacuum concentrator (45 °C, > 1 h) after separating each phase into different vials. To analyse the largest number of metabolites, both phases would be analyzed. First, the aqueous extract was re-suspended using deuterated water (D2O) containing trimethylsilyl-propanoic acid (TSP), and then later the mixture was vortexed followed by centrifugation at 12,000 g for 5 min. 550 µL of the supernatant was transferred into an NMR tube. Second, the lipophilic metabolites extract was re-suspended in 580 µL of deuterated chloroform (CDCl_3_) containing 0.03 vol/vol TMS. The mixture was centrifuged at 1000 × g, for 5 min and then 550 µL of the supernatant was transferred into an NMR tube. The tubes were subjected to ^1^H-NMR analysis^10^.

### Total RNA extraction and gene expression levels quantified with real-time PCR analysis

It is well known that glucose is absorbed by liver cells in response to insulin. Additionally, it has been shown that insulin has an impact on the brain and plays an important role in diabetes. Thus, the targeted genes were insulin receptors (A and B), AKT, FoxO1a, and GPR151 in both liver and brain of the tested zebrafish. The total RNA extraction, cDNA synthesis, and RT-PCR were performed following the Quick-start protocol RNeasy plus mini kit, QuantiTect reverse transcription kit, and QuantiNova SYBR green PCR kit, respectively (Qiagen^®^, Hilden, Germany). Table 1 contains the used primers for each gene.

**Table 1:** Primer’s sequence of target genes.

Targeted genes	Forward (5′-3′) Revers (3′-5′)	References
AKT2-F	5′ AACAGAGGCTTGGCGGAGGT 3′	^11^
AKT2-R	3′ TCCTCCGCGTCAAGACTGTCA 5′	
Insulin receptor a-F	5′ GGAGCCCCACTCGTCTAACAAA 3′	^12^
Insulin receptor a-R	3′ CGCCGTTGTGAATGACGTATTC 5′	
Insulin receptor b-F	5′ GACTGATTACTATCGCAAGGG 3′	
Insulin receptor b-R	3′ TCCAGGTATCCTCCGTCCAT 5′	
FoxO1a-F	5′ GCGGCAAAGAAAAAGCTGGC 3′	^13^
FoxO1a-R	3′ TCATTGCTGTGGGAGTTCGG 5′	
GPR151-F	5′ CCTAAACAAGAAGCTACCATCTGCA 3′	^14^
GPR151-R	3′ AGTCAGAGGACTTGCAGATGAACC 5′	
GAPDHA-F	5′ GAGCACCGTTCATGCTATC 3′	^15^
GAPDHA-R	3′ GACCATCCCTCCACAGTTTT 5′	

### Statistical analysis

SPSS statistics 25 (IBM^®^, Chicago, USA) was used to display the data as mean standard deviation (SD). The *P*-value was determined using the ANOVA analysis and the Tukey's test, with a significance level of *P* < 0.05 deemed significant. After binning the NMR spectra, MVDA via principal component analysis (PCA), partial least square (PLS) regression, and the supervised orthogonal projection to latent structures-discriminant analysis (OPLS-DA) were conducted using the Parreto scaling method in SIMCA-P software (Umetrics^®^ v. 14.0, Ume, Sweden). The variable in the created data matrix was the NMR chemical shifts, and the observations were the sample names. The findings of the bioassay are expressed as the means with a standard deviation. A two-way analysis of variance (ANOVA) was used to determine the significance of the difference between the bioassay data obtained at a 95% confidence interval. All analyses were conducted using the statistical program SPSS statistics 25. The relevant ^1^H NMR signals were allocated from the binned data after Chenomx processing and were chosen based on their distinct properties that did not overlap with those of other substances.

### Ethical approval

The experimental procedures were approved by the Commission of Ethics at Monash University, Malaysia, the research was conducted following the internationally accepted principles for laboratory animal use and care as found in the European Community guidelines (EEC Directive of 1986; 86/609/EEC).

## Results

### Induction of diabesity in adult zebrafish

**Figure 1:**
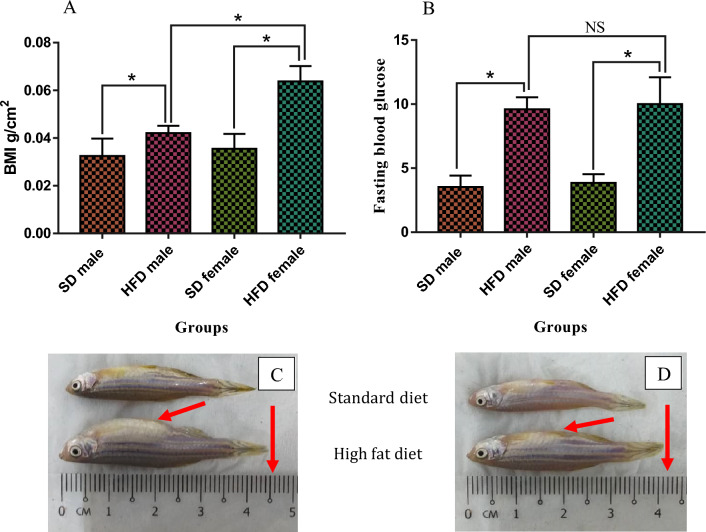
BMI (g/cm^2^) and fasting blood glucose level (mmol/L) of zebrafish following over-feeding with HFD and standard diet for 4 weeks. Values are means (± SD), (*P) indicates a significant difference (*P* < 0.05) among the values, with n = 8. *NS* no significant difference, *SD* standard diet, *HFD* high-fat diet. Exemplary images of zebrafish (C: female; D: male) were included in each of the analyzed dietary groups at week 4 of feeding. Red arrows point to the abdominal region. Overfeeding with the high-fat diet of zebrafish showed a significant increase in weight and length.

Figure 1A shows that the BMI (g/cm^2^) of male and female zebrafish following an HFD for 4 weeks was greater and significantly different (*P* < 0.05) from that of males and females following an SD over the same duration. The BMI of HFD males (0.042 g/cm^2^) was significantly different (*P* > 0.05) from that of HFD females (0.063 g/cm^2^). Figure 1B shows that the fasting blood glucose (mmol/L) levels in males and females on an HFD are substantially higher and statistically different (*P* < 0.05) from those on an SD. Figure 1C and D gives exemplary images of male and female zebrafish in each of the analyzed dietary groups at week 4 of feeding. The red arrows point to the abdominal region and the length of the HFD zebrafish which showed a larger size compared to the SD zebrafish.

### Oral glucose tolerance test (OGTT)

**Figure 2:**
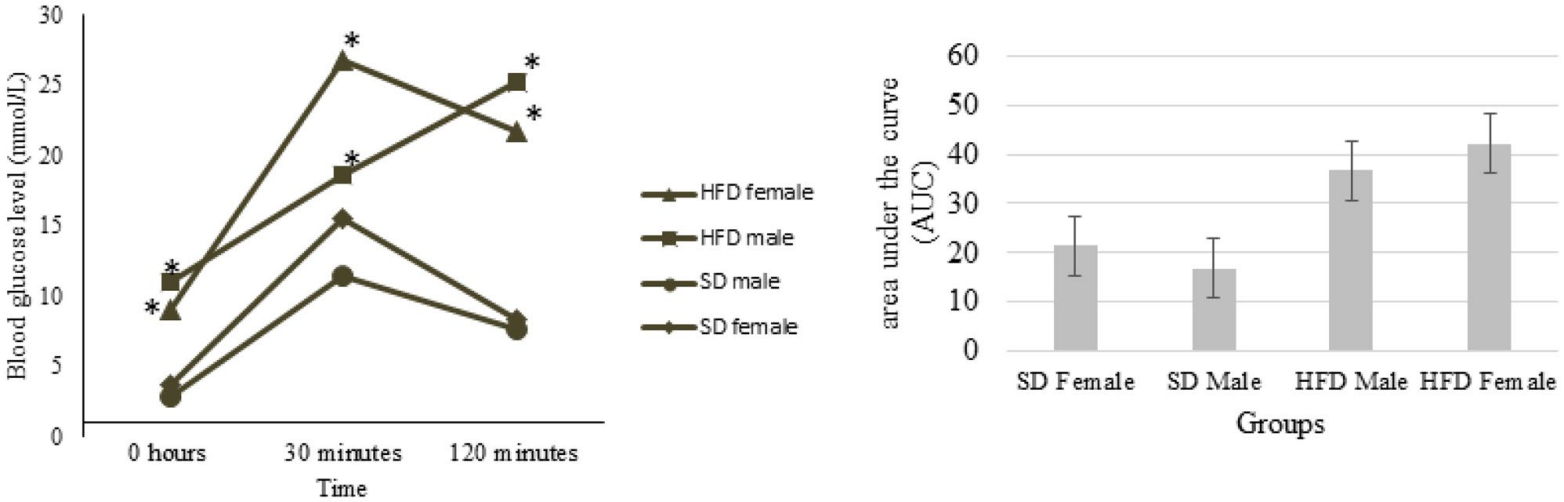
Glucose tolerance test (GTT) and area under the curve (AUC) of male and female zebrafish fed an HFD versus SD. Blood glucose levels were measured after fasting for 0, 30, and 120 min, and following an oral dose of 1.25 mg glucose/g body weight; Values are means (± SD), (*P) indicates significant difference (*P* < 0.05) among the values, n = 8 in each time point. SD: standard diet. HFD: high-fat diet.

To corroborate the creation of the model, the OGTT test was conducted. Figure 2 demonstrates that blood glucose (mmol/L) levels following oral glucose administration (1.25 mg/g body weight) in males and females on an HFD were significantly higher and statistically different (*P* < 0.05) to those on an SD at all time points examined (0, 30, 120 min). There is no significant difference (*P* > 0.05) in blood glucose levels between males and females on an SD at the three time points. Additionally, the blood glucose levels of males on the HFD are substantially different from those of females on the HFD at 0 and 30 min. However, there is no significant difference (*P* > 0.05) between males and females on HFD at 120 min.

### Treatment of T2D zebrafish with DL-limonene

**Figure 3:**
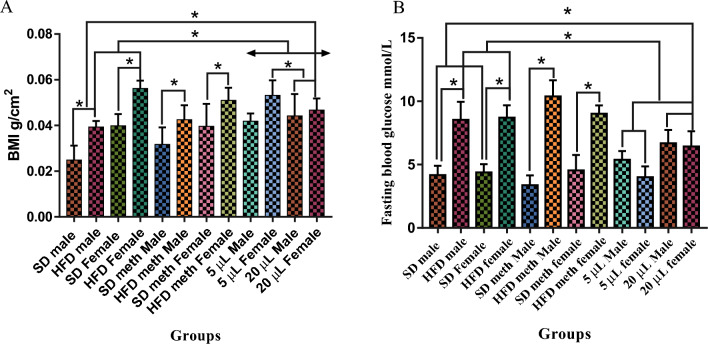
BMI (**A**) (g/cm^2^) and fasting blood glucose level (**B**) (mmol/L) of non-treated and treated male and female zebrafish with different concentrations of DL-limonene following HFD and SD. Values are means (± SD), (*P) indicates a significant difference (*P* < 0.05) among the values, n = 6. SD: standard diet. *HFD* High-fat diet.

Figure 3A shows the BMI (g/cm^2^) of males and females of SD, HFD and HFD treated with 5 and 20 mg/L of DL-limonene for one month. The BMI of untreated HFD male (0.039 (± 0.58) g/cm^2^) and female (0.056 (± 0.40) g/cm^2^) zebrafish are significantly different (*P* > 0.05) from that of the respective male (0.024 (± 0.29) g/cm^2^ and female (0.039 (± 0.26) g/cm^2^) SD zebrafish. The BMI of groups treated with 5 mg/L DL-limonene is significantly lower than any of the other groups (HFD, HFD treated with methanol, and HFD treated with 20 mg/L of DL-limonene). The BMI of the males (0.044 (± 0.42) g/cm^2^) and females (0.046 (± 0.48) g/cm^2^) in the group treated with 20 mg/L is significantly different from the SD group, HFD females, HFD treated with methanol groups and treated groups with 5 mg/L DL-limonene.

Figure 3B shows the fasting blood glucose (mmol/L) of males and females of SD, HFD, and HFD groups treated with methanol and 5 and 20 mg/L of DL-limonene for one month. The fasting blood glucose of HFD males (8.53 (± 0.58) mmol/L) and HFD females (8.7 (± 0.40) mmol/L) is higher and significantly different from that of SD groups. Similarly, the fasting blood glucose of HFD females (9.02 (± 0.26) mmol/L) and HFD males treated with methanol (10.38 (± 0.51) mmol/L) is significantly higher than the SD groups. The fasting blood glucose of HFD males (6.68 (± 0.42) mmol/L) and females (6.43 (± 0.48) mmol/L) treated with 20 mg/L is significantly higher than the SD but it is significantly lower than the HFD female groups. However, the fasting blood glucose of HFD males treated with 20 mg/L shows no significant difference (*P* > 0.05) from the HFD males. There is no significant difference (*P* > 0.05) between males and females of HFD treated with 20 mg/L of DL-limonene.

### Analysis of HFD compared to SD and biomarkers identification of diabesity zebrafish

**Figure 4:**
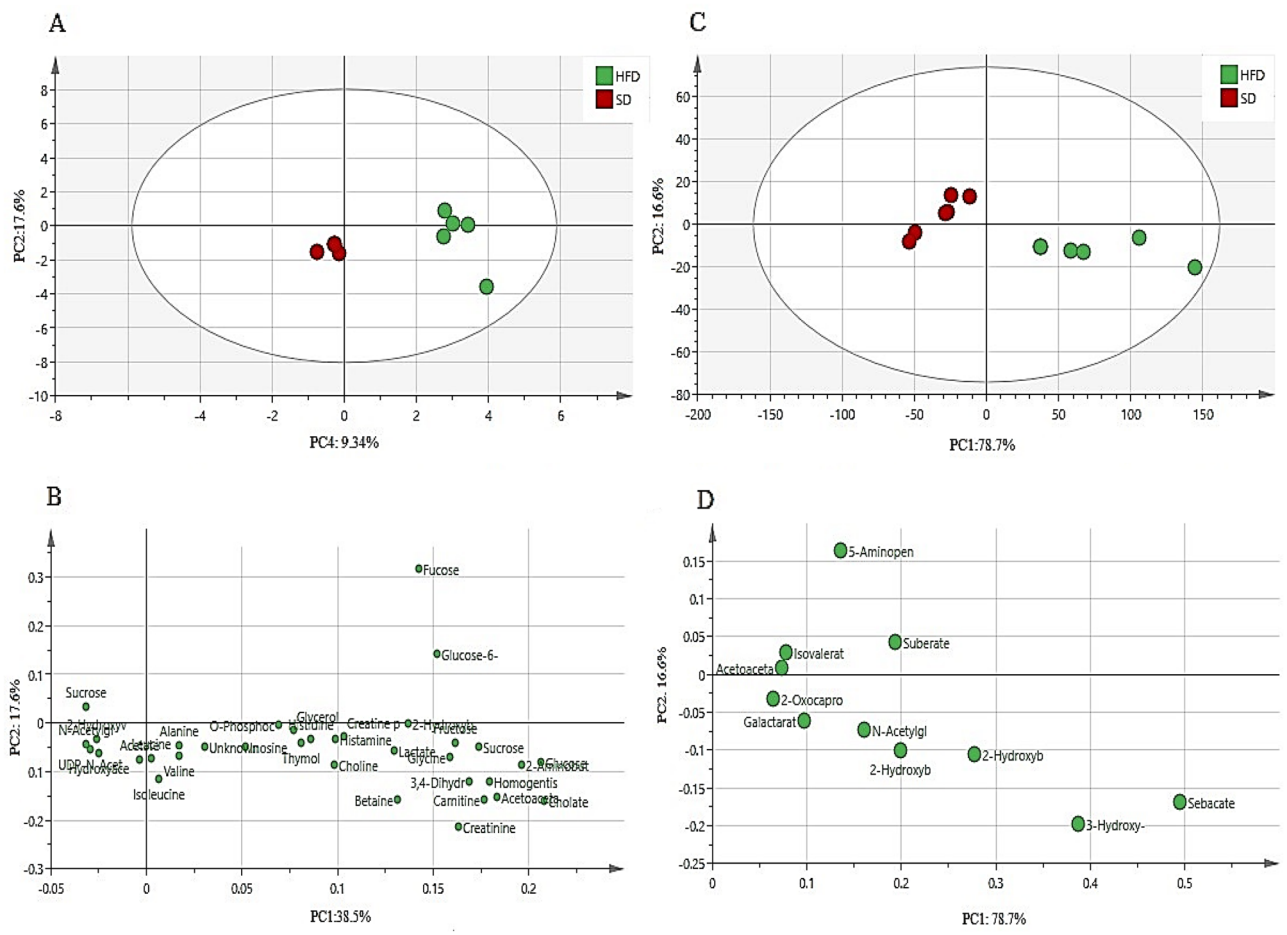
The PCA score (**A** and **C**) and loading (**B** and **D**) plots (PC1 vs. PC2) compare the HFD with the SD male zebrafish of aqueous and lipophilic metabolites after 4 weeks of treatment. *HFD* high fat diet, *SD* stander diet.

The PCA score plot (Fig. 4A and C) revealed a clear separation by PC1 between the HFD and SD male zebrafish in both aqueous and lipophilic metabolites. The primary difference in the HFD group was the increased level of all lipophilic metabolites including sebacate, 3-hydroxy-3-methylglutarate, 2-hydroxybutyrate, 2-hydroxyisovalerate, and suberate. For aqueous metabolites, the content of sucrose, glucose-6-phosphate, fructose, glucose, and fucose was increased. However, some other aqueous metabolites decreased including isoleucine, betaine, 2-hydroxybutyrate, valine, and creatinine (Fig. 4B and D).

**Figure 5:**
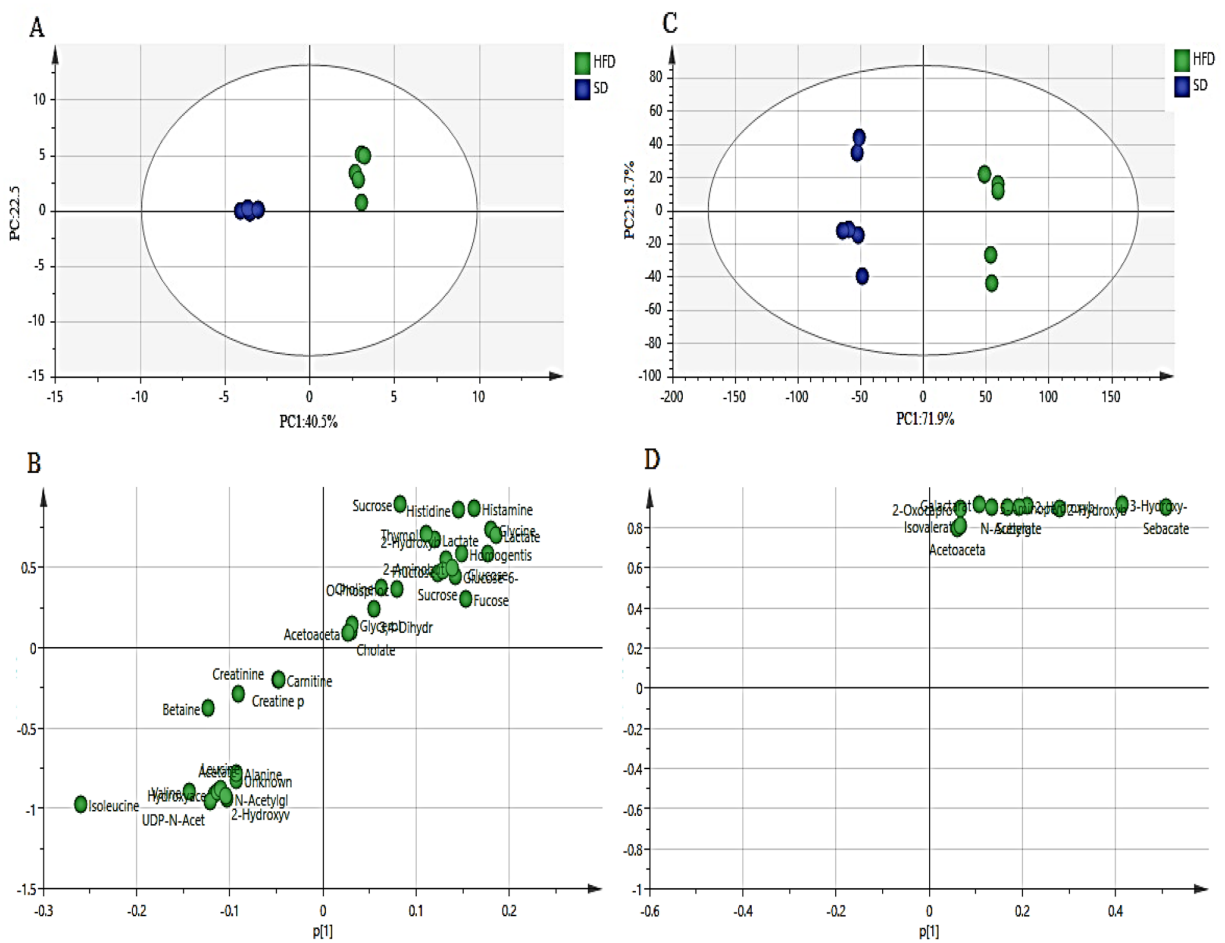
The OPLS-DA score plot (**A** and **C**) and S-plot (**C** and **D**) of aqueous (**A** and **B**) and lipophilic (**C** and **D**) metabolites of HFD and SD male zebrafish after 4 weeks of treatment. *HFD* high fat diet, *SD* stander diet.

The supervised orthogonal projection to latent structures-discriminant analysis (OPLS-DA) was also used to iddentify the significant aqueous and lipophilic metabolites related to the SD and HFD male zebrafish as well as to discover biomarkers associated with HFD. The OPLS-DA score plot (Fig. 5A and C) showed a clear separation between the SD and HFD groups in both aqueous and lipophilic metabolites. The S-plot is the OPLS-DA model’s covariance and correlation loading diagnostic, providing an overview of the model’s influencing factors and filtering relevant metabolites in the projection (Fig. 5B and D). The metabolites that are considerably upregulated in the HFD are in the upper-right quadrant of the S-plot, which has positive correlations and covariances. The decreasing ones, on the other hand, are in the lower-left quadrant. The metabolites of HFD male zebrafish were identified based on the chemical shift data of the standard compounds available in the Chenomx library. All the lipophilic metabolites were raised including sebacate, 3-hydroxy-3-methylglutarate, 2-hydroxybutyrate, 2-hydroxyisovalerate, suberate, n-acetylglutamate, 5-aminopentanoate, galactarate, 2-oxocaproate, isovalerate, and acetoacetate. However, the aqueous metabolites that show an increase are histamine, histidine, sucrose, glucose-6-phosphate, fructose, lactate, 2-hydroxyvalerate, glucose, fucose, 2-aminobutyrate, glycerol, homogentisate, cholate, 3,4-dihydroxybenzeneacetate, lactate, acetoacetate, choline, thymol, inosine, glycine, and *O*-phosphocholine. Meanwhile, other aqueous metabolites were decreased including isoleucine, betaine, 2-hydroxybutyrate, valine, creatinine, udp-n-acetylglucosamine, hydroxyacetone, acetate, leucine, creatine-phosphate, *N*-acetylglutamate, carnitine, and alanine.

**Figure 6:**
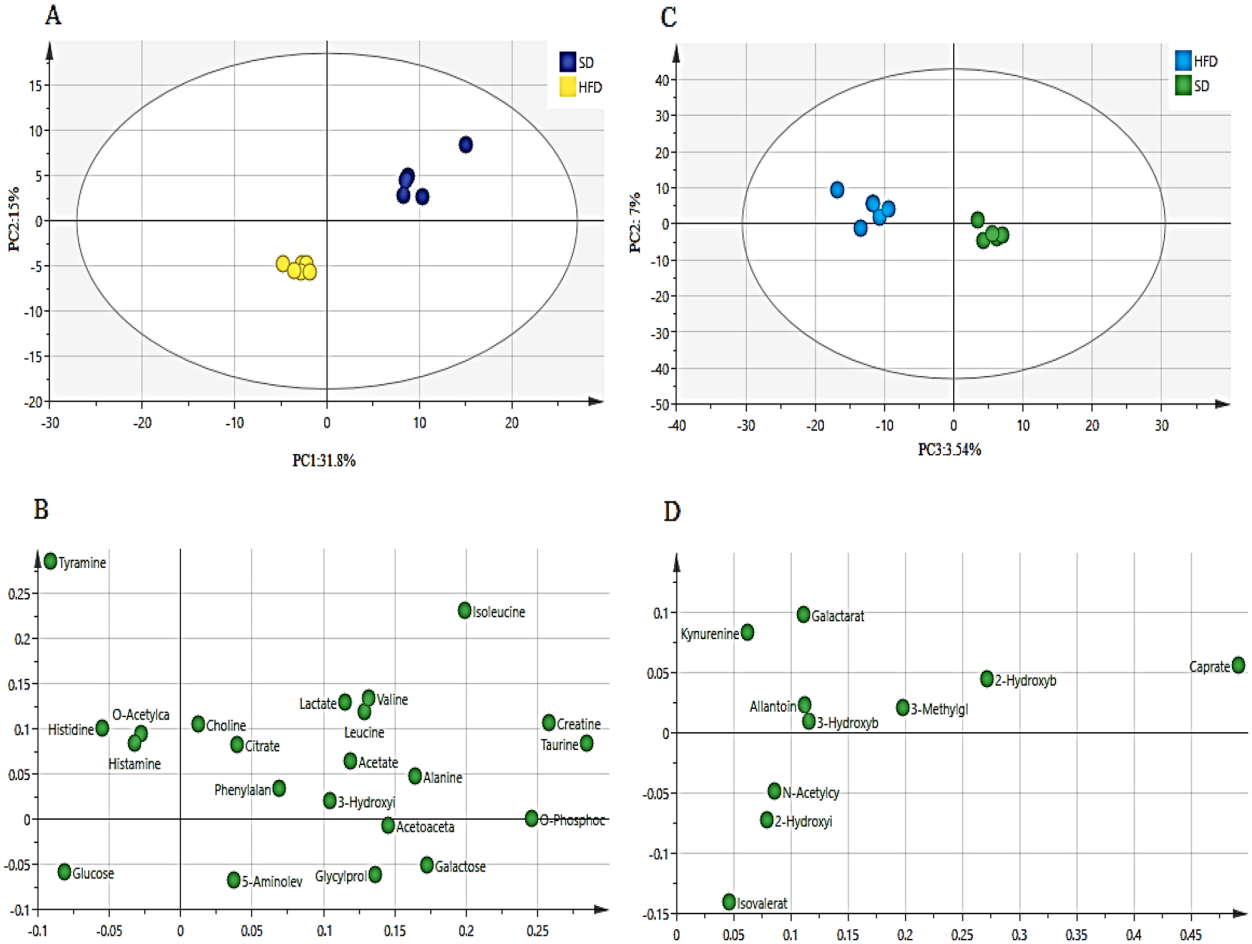
The PCA score (**A** and **C**) and loading (**B** and **D**) plots (PC1 vs. PC2) compare the HFD with the SD female zebrafish of aqueous and lipophilic metabolites after 4 weeks of treatment. *HFD* high fat diet, *SD* stander diet.

The same trend was observed for the female fish. The PCA score plot (Fig. 6A and C) revealed a significant difference between the HFD and SD female zebrafish in both aqueous and lipophilic metabolites. The primary variations in the HFD group were the raised level of all detected lipophilic metabolites including caprate, 2-hydroxybutyrate, 3-methylglutarate, 3-hydroxybutyrate, galactarate, allantoin, n-acetylcysteine, isovalerate, kynurenine, and 2-hydroxyisocaproate. Aqueous metabolites including isoleucine, creatinine, taurine, leucine, and acetate were decreased, while glucose, histamine, histidine, and tyramine were increased (Fig. 6B and D).

**Figure 7:**
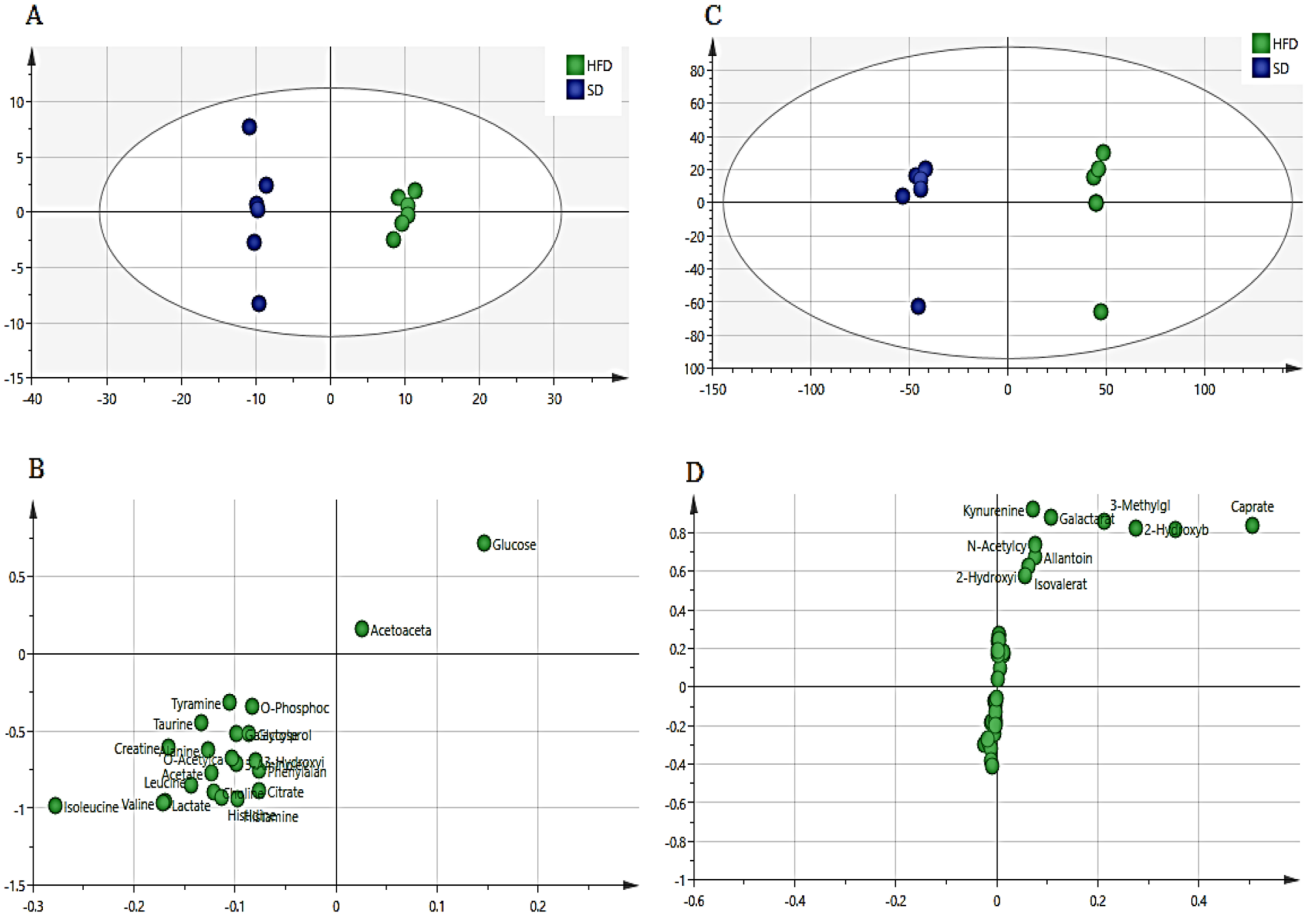
The OPLS-DA score plot (**A** and **C**) and S-plot (**C** and **D**) of aqueous (**A** and **B**) and lipophilic (**C** and **D**) metabolites of HFD and SD female zebrafish after 4 weeks. *HFD* high fat diet, *SD* stander diet.

The supervised orthogonal projection to latent structures-discriminant analysis (OPLS-DA) was also used to show unambiguous findings of the significant aqueous and lipophilic metabolites related to the SD and HFD female zebrafish. The OPLS-DA score plot (Fig. 7A and C) showed a clear separation between the SD and HFD groups in both aqueous and lipophilic metabolites. The S-plot is the OPLS-DA model's covariance and correlation loading diagnostic, providing an overview of the model's influencing factors and filtering relevant metabolites in the projection (Fig. 7B and D). The metabolites that are considerably enhanced in the HFD are in the upper-right quadrant of the S-plot, which has positive correlations and covariances. The decreasing ones, on the other hand, are in the lower-left quadrant.

### Metabolic changes in diabesity zebrafish treated with DL-limonene

**Figure 8:**
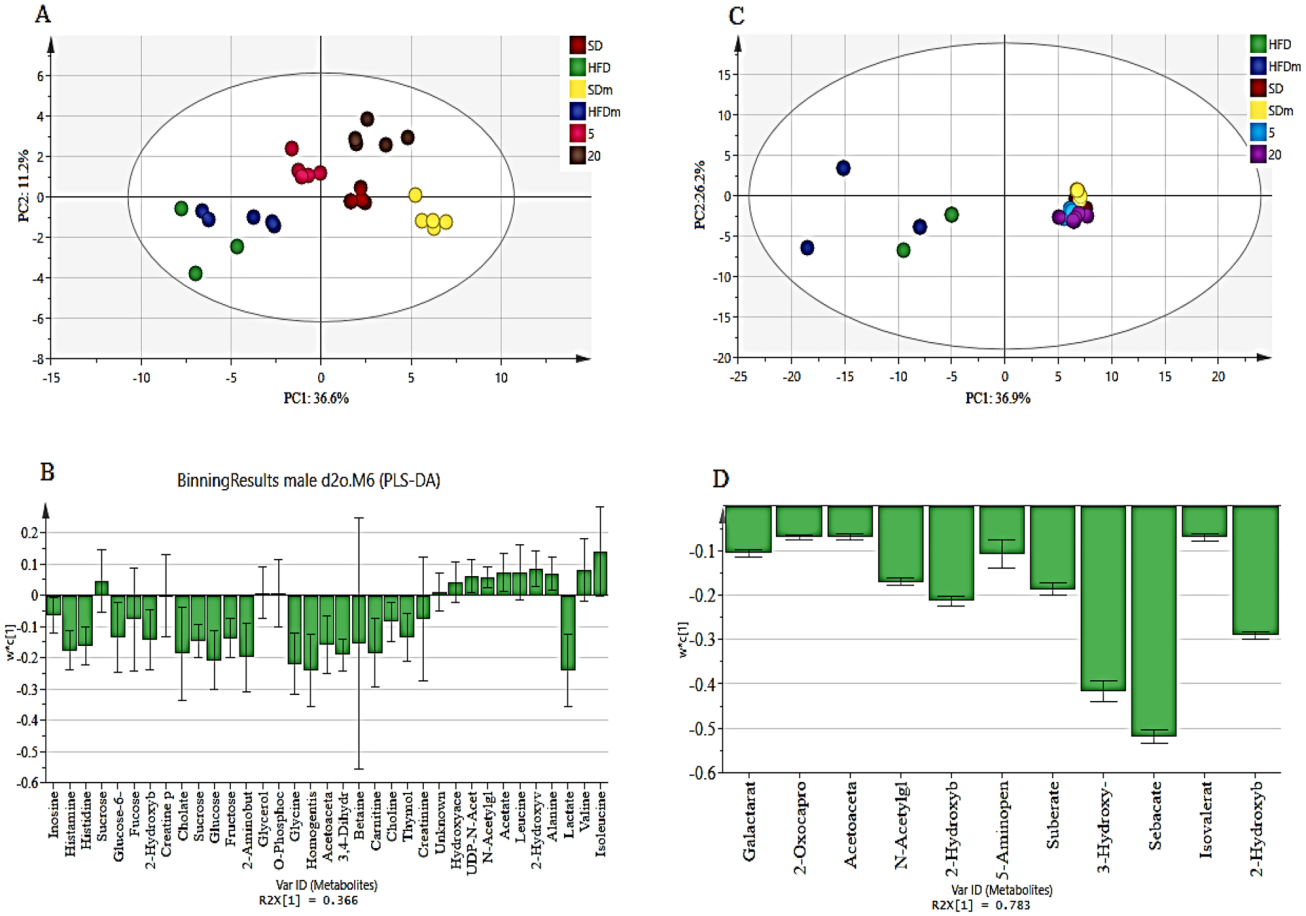
The PLS-DA score (**A** and **C**) and loading column (**B** and **D**) plots of the aqueous metabolites (**A** and **B**) and lipophilic metabolites (**C** and **D**) show the effects of low and high doses (5 and 20 mg/L) of DL-limonene on male diabesity zebrafish after 4 weeks of treatment.

**Figure 9:**
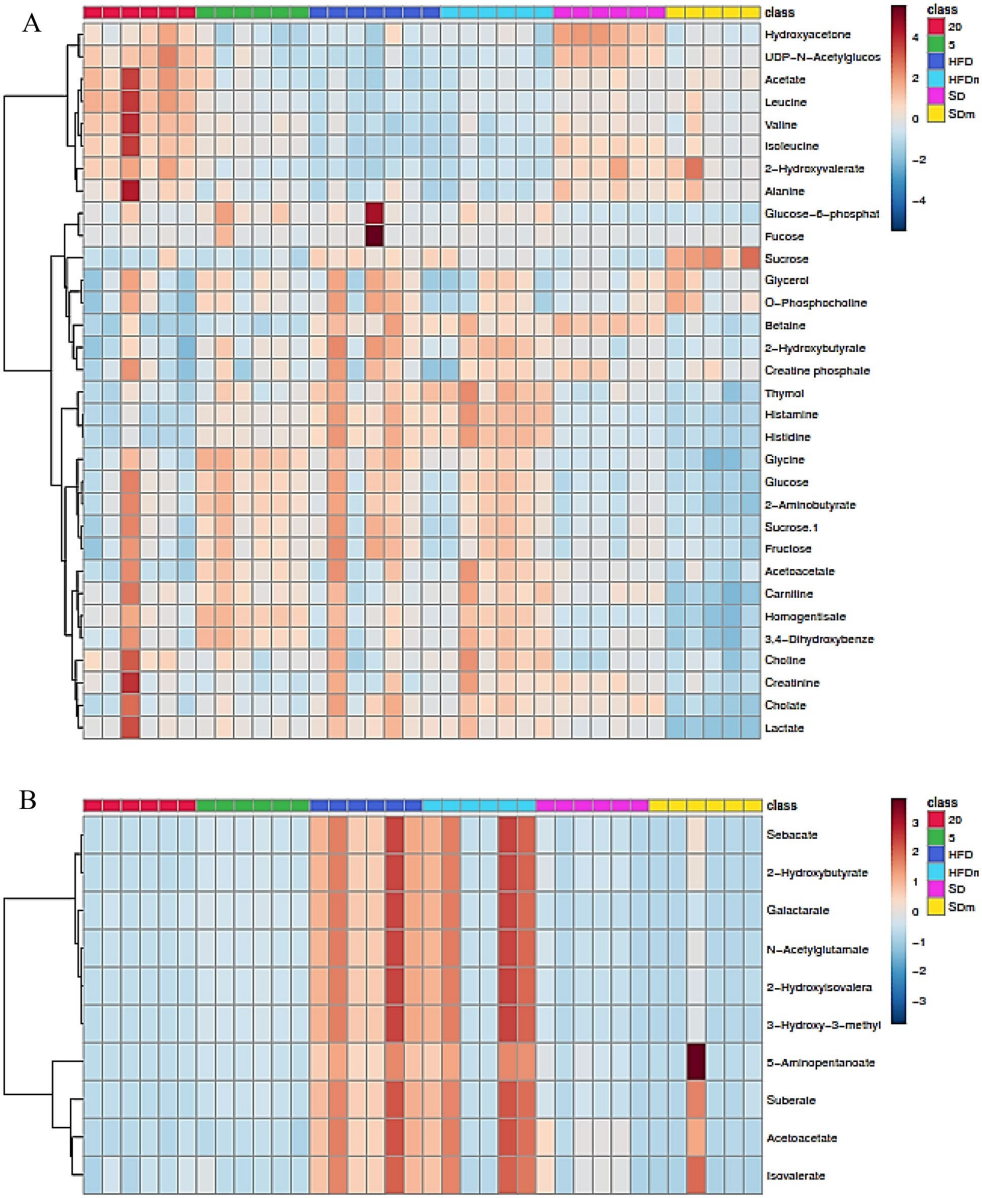
The heatmap of aqueous (**A**) and lipophilic (**B**) metabolites showing the effects of low and high doses of DL-limonene on the treated obese diabesity male zebrafish after 4 weeks of treatment. *HFD* high fat diet, *SD* stander diet, *20* 20 mg/L, *5* 5 mg/L, *HFDm* high fat diet methanol, *SDm* stander diet methanol.

**Figure 10:**
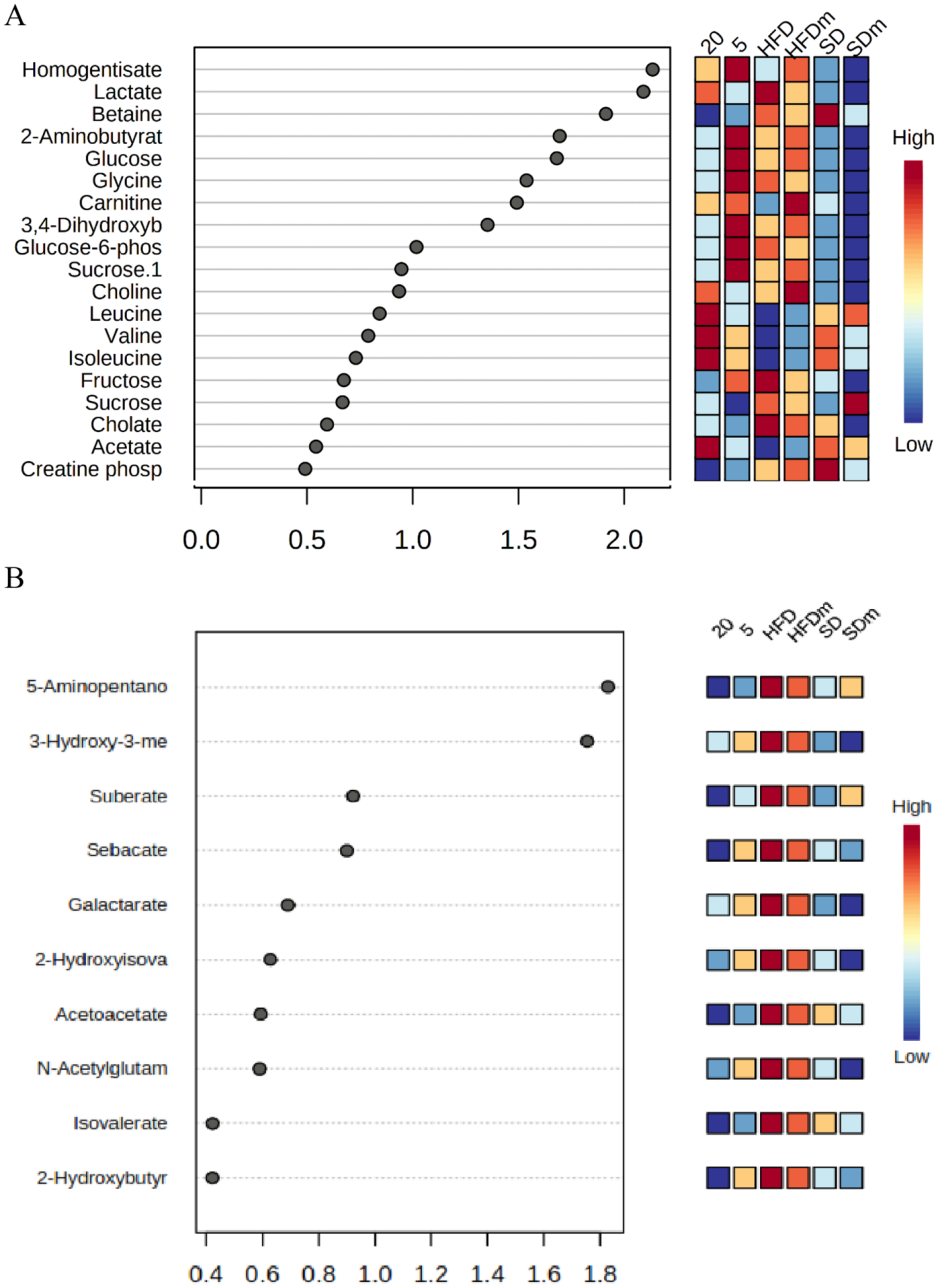
The VIP values were derived from PLS-DA of aqueous (**A**) and lipophilic (**B**) metabolites of diabesity male zebrafish. *HFD* high fat diet, *SD* stander diet, *20* 20 mg/L, *5* 5 mg/L, *HFDm* high fat diet methanol, *SDm* stander diet methanol.

A supervised projection to partial least squares-discriminant analysis (PLS-DA) was applied to identify the significant aqueous and lipophilic metabolites associated with the HFD and HFD treated group of male zebrafish (Fig. 8). Both PC 1 and 2 described a total variance of 73.8% with high-class discriminant R2Y and Q2 values of 41.6 and 32.2, respectively, indicating that the suggested aqueous metabolites were significantly affected by the treatment. However, both R2Y (98.8) and Q2 (96) values of the lipophilic metabolites were higher than the aqueous metabolites indicating higher class discriminant. The score plot shows a clear separation where the HFD groups are clustered separately on the left side of the score plot. Meanwhile, the treated groups with DL-limonene cluster is found on the right side, close to the SD groups. The loading column (Fig. 8B and D) shows the variability of the biomarkers in the treated groups which leads to the separation from the HFD groups. Treatment with DL-limonene helped to reduce all the lipophilic metabolites including galactarate, 2-oxocaproate, acetoacetate, *N*-acetylglutamate, 2-hydroxybutyrate, 5-aminopentanoate, suberate, 3-hydroxy-3-methylglutarate, sebacate, isovalerate, and 2-hydroxybutyrate. The DL-limonene treatment increased aqueous metabolites including valine, isoleucine, alanine, 2-hydroxyvalerate leucine, acetate, UDP-*N*-acetylglucosamine, hydroxyacetone, *O*-phosphocholine, glycerol, and sucrose. However, the metabolites inosine, histamine, histidine, glucose-6-phosphate, fucose, 2-hydroxybutyrate, creatine phosphate, cholate, sucrose, glucose, fructose, 2-aminobutyrate, glycine, homogentisate, acetoacetate, 3,4-dihydroxybenzeneacetate, betaine, carnitine, choline, thymol, creatinine, and lactate were decreased. The metabolomics pattern of the key metabolites of male diabesity zebrafish was expressed as squares in a heatmap. These squares represent the metabolites and their contents are indicated by colours based on a normalized scale from − 4 (low) to 4 (high) in aqueous metabolites and from − 3 (low) to 3 (high) in lipophilic metabolites as shown in (Fig. 9). These analyses were consistent with the separation of the animal model into different groups based on the PLS-DA. To identify the most significant contributing variables, the VIP analysis of the PLS-DA model was considered (Fig. 10).

**Figure 11:**
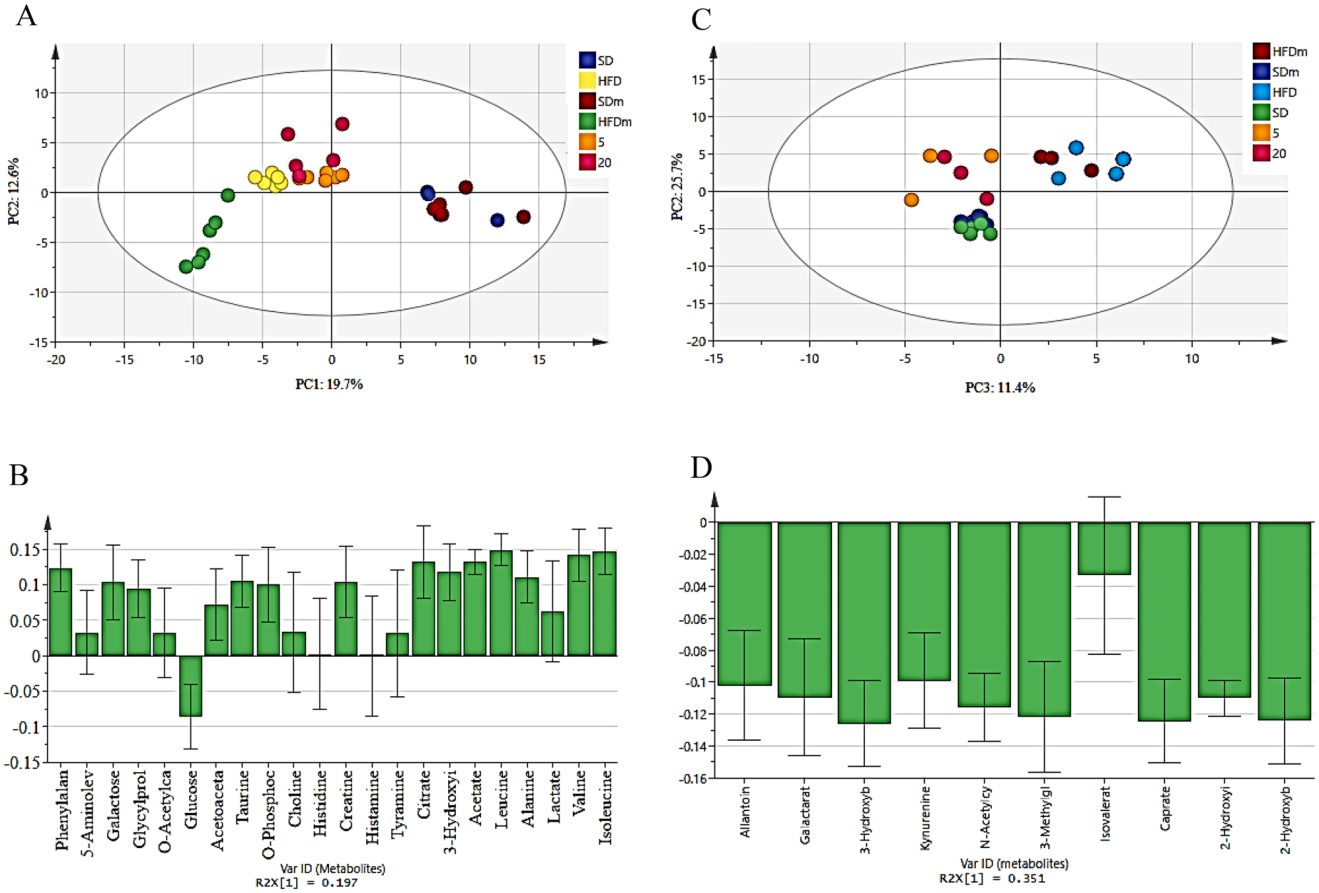
The PLS-DA score (**A** and **C**) and loading column (**B** and **D**) plots of the aqueous metabolites (**A** and **B**) and lipophilic metabolites (**C** and **D**) show the effects of low and high doses (5 and 20 mg/L) of DL-limonene on female diabesity zebrafish after 4 weeks of treatment.

**Figure 12:**
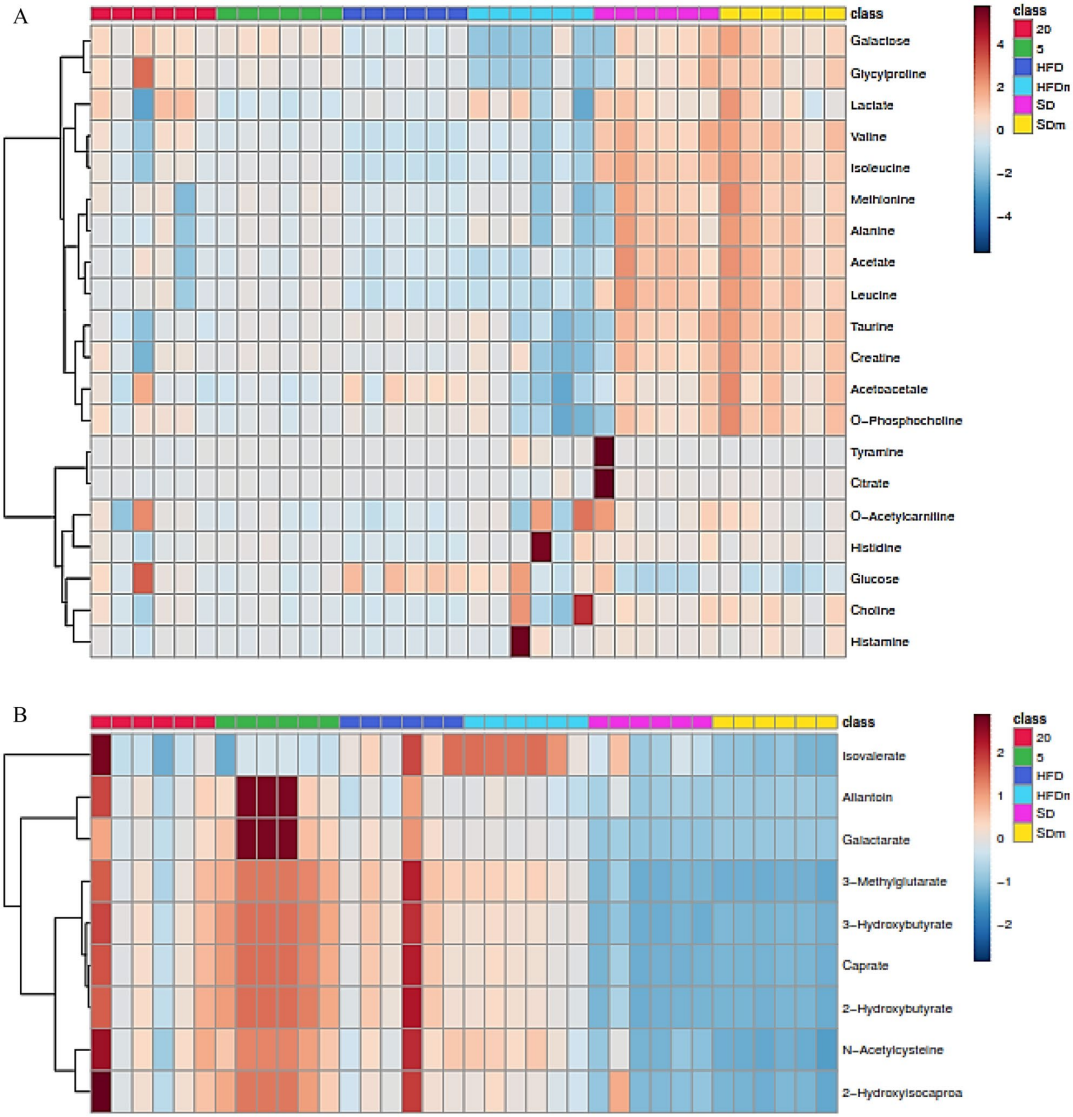
The heatmap of aqueous (**A**) and lipophilic (**B**) metabolites showing the effects of low and high doses of DL-limonene on the treated diabesity female zebrafish after 4 weeks of treatment. *HFD* high fat diet, *SD* standard diet, *20* 20 mg/L, *5* 5 mg/L, *HFDm* high fat diet methanol, *SDm* standard diet methanol.

**Figure 13:**
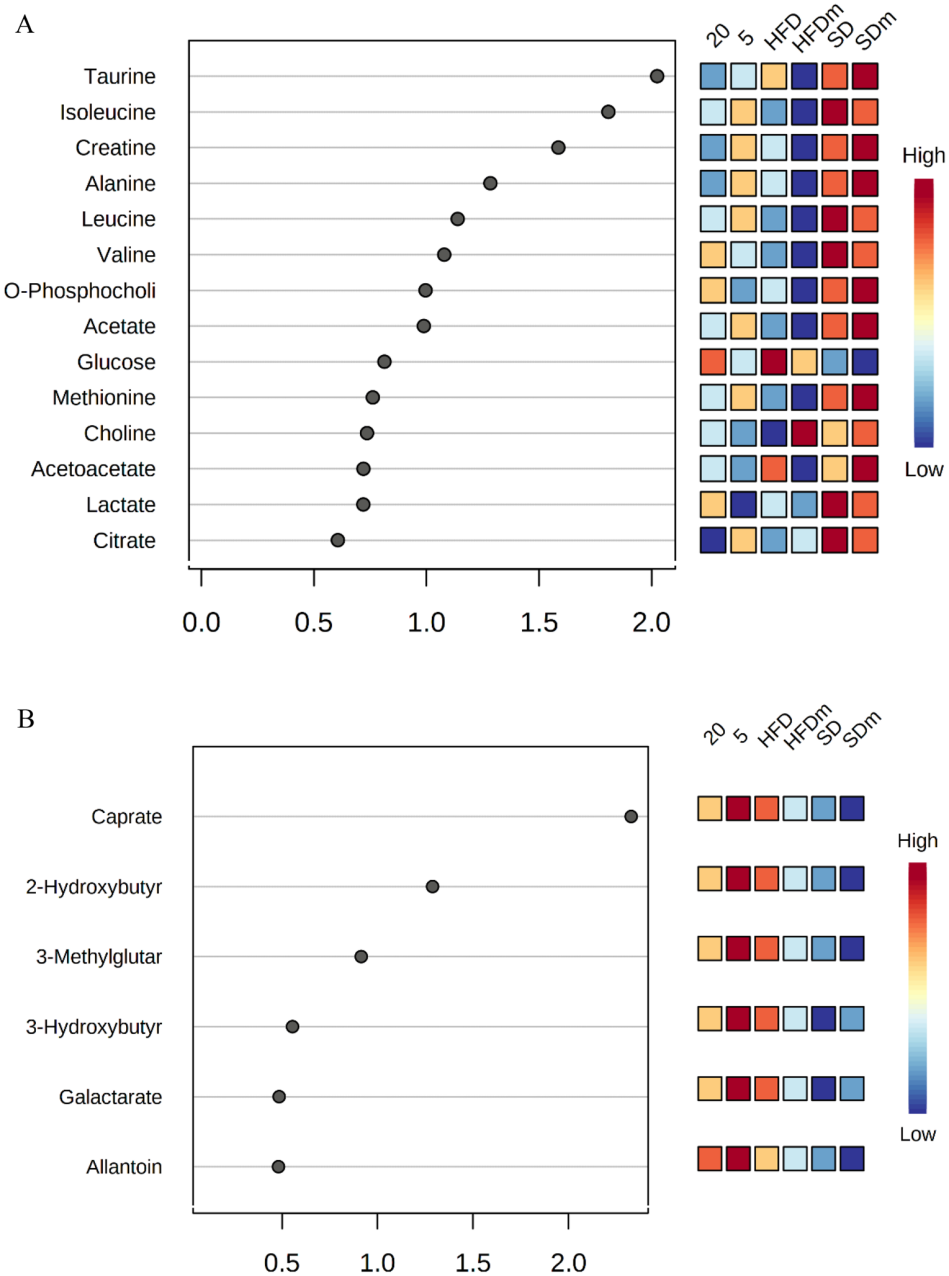
The VIP values were derived from PLS-DA of aqueous (**A**) and lipophilic (**B**) metabolites of diabesity female zebrafish. *HFD* high fat diet, *SD* standard diet, *20* 20 mg/L, *5* 5 mg/L, *HFDm* high fat diet methanol, *SDm* standard diet methanol.

A supervised projection to PLS-DA was also applied to identify the significant aqueous and lipophilic metabolites associated with the HFD and treatment with DL-limonene in female zebrafish. Both PC 1 and 2 described a total variance of 37.1% with high-class discriminant R2Y and Q2 values of 60.2 and 47.7, respectively. The score plot shows that the treated groups in the aqueous metabolites of female zebrafish (Fig. 11A) are clustered with the HFD groups, however, with a shift toward to the SD groups. However, the lipophilic metabolites score plot (Fig. 5C) shows a better separation of the treated groups where it clusters with the SD groups. The R2Y (78) and Q2 (78.1) values of the lipophilic metabolites were higher than the aqueous metabolites indicating a higher-class discriminant. The loading column (Fig. 11B and D) shows the variability of the biomarkers in the treated groups which lead to the separation from the HFD groups. Treatment with DL-limonene helped to reduce all the lipophilic metabolites including allantoin, galactarate, 3-hydroxybutyrate, kynurenine, *N*-acetylcysteine, 3-methylglutarate, isovalerate, caprate, 2-hydroxyisocaproate, and 2-hydroxybutyrate. Glucose and histamine were the only aqueous metabolite that shows a decrease, while, the rest of the aqueous metabolites were increased including phenylalanine, 5-aminolevulinate, galactose, glycylproline, *O*-acetylcarnitine, acetoacetate, taurine, *O*-phosphocholine, choline, histidine, creatine, tyramine, citrate, 3-hydroxyisovalerate, acetate, leucine, alanine, lactate, valine, and isoleucine. The metabolomics pattern of the key metabolites of female diabesity zebrafish was expressed as squares in a heatmap. These squares represent the metabolites and their contents are indicated by colors based on a normalized scale from − 4 (low) to 4 (high) in aqueous metabolites and from − 2 (low) to 2 (high) in lipophilic metabolites (Fig. 12). These analyses were consistent with the separation of the animal model into different groups based on the PLS-DA. To identify the most significant contributing variables, the VIP analysis of the PLS-DA model was considered (Fig. 13).

### Metabolic pathway analysis of diabetic zebrafish.

**Figure 14:**
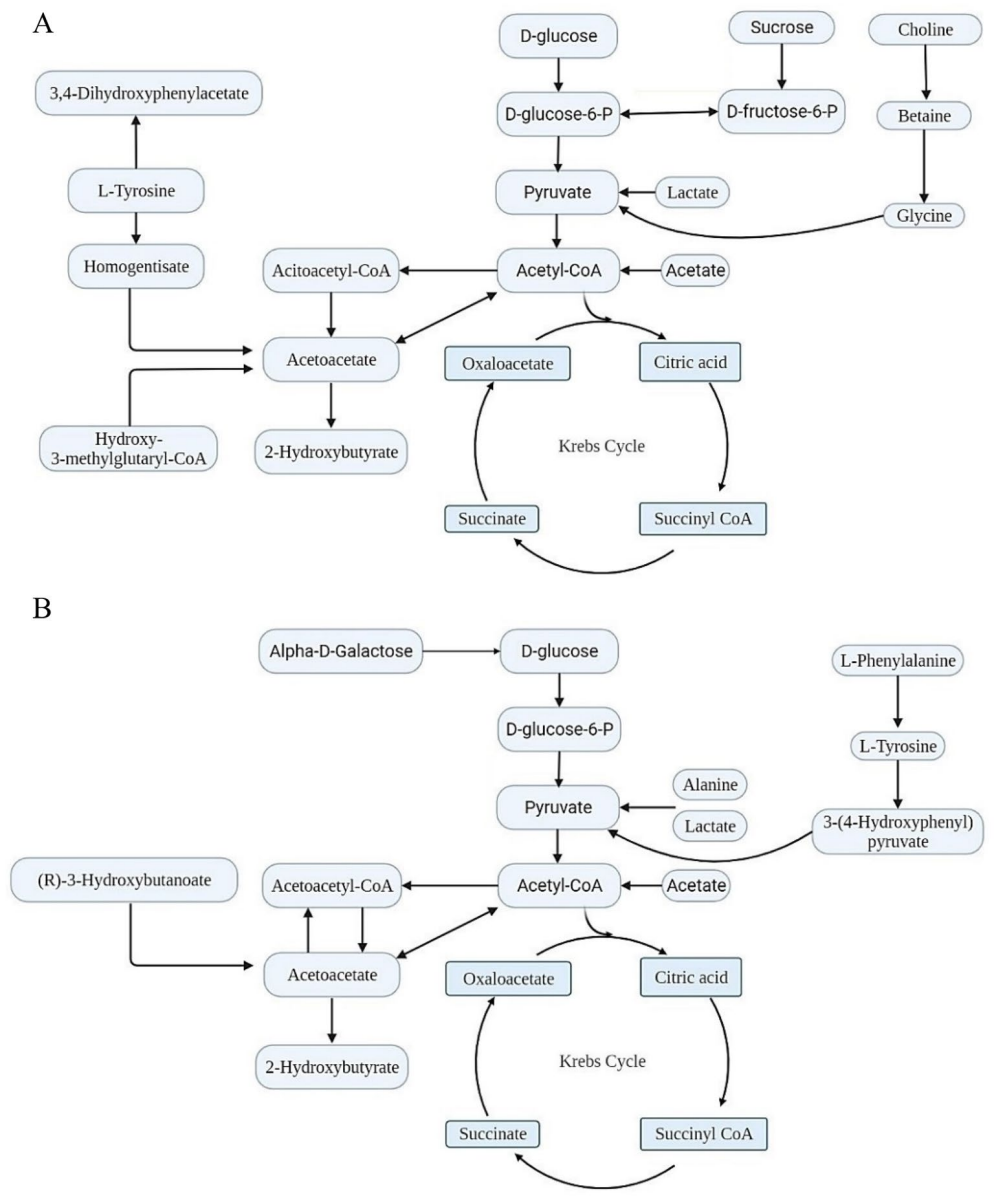
Metabolic pathways and metabolite changes observed in diabetic (**A**) male and (**B**) female (**B**) zebrafish.

Figure 14 shows the most significant pathways triggered by the HFD in male and female diabesity zebrafish. The identified metabolites were submitted to MetaboAnalyst online software for pathway detection analysis.

### Total RNA extraction and gene expression levels quantified with real-time PCR analysis

**Figure 15:**
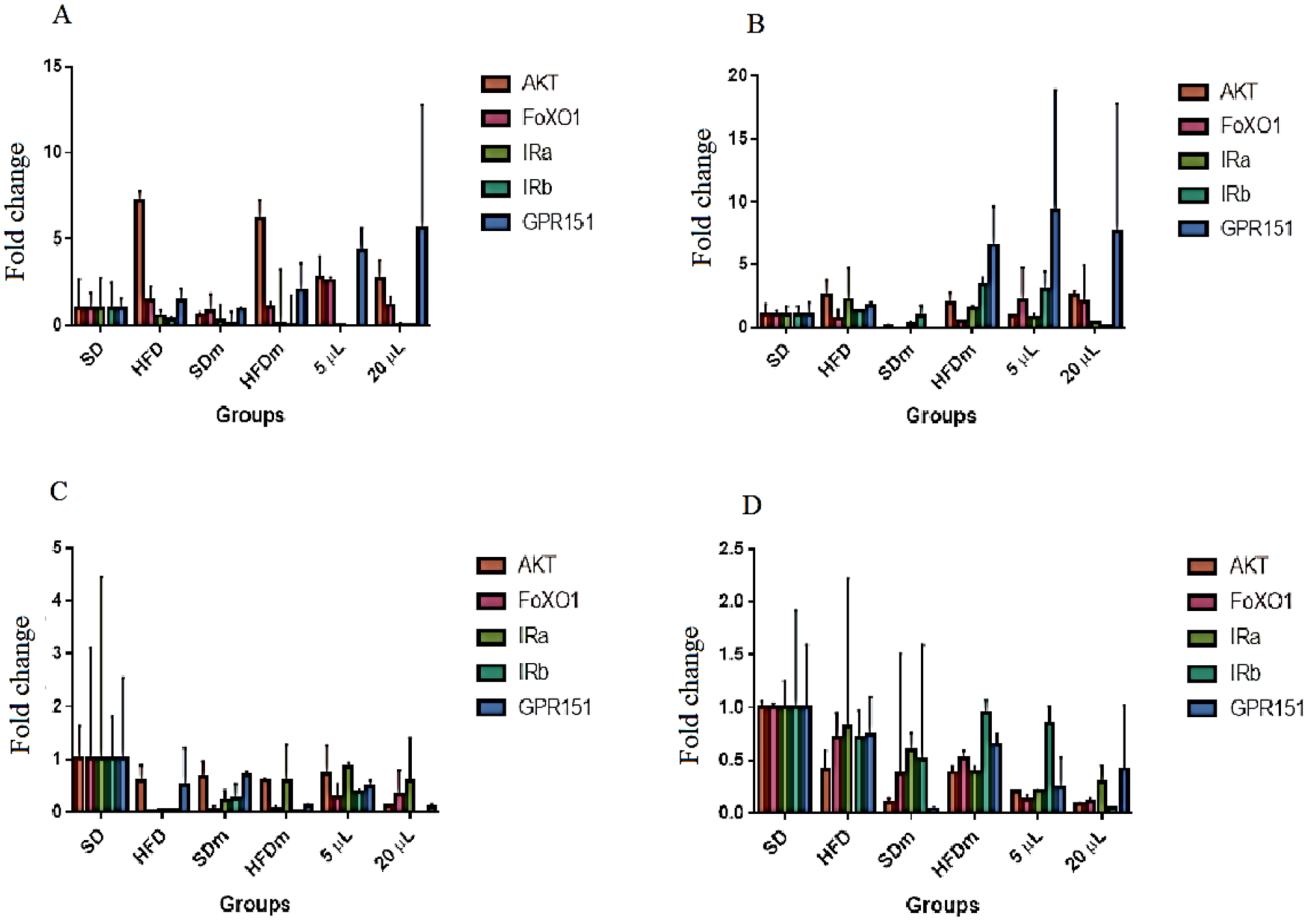
The foldchange of the tested liver (**A**: Female; **B**: Male) and brain (**C**: Female; **D**: Male) genes.

Among the female liver groups, the housekeeping gene GAPDH had a Ct value mean ranging between 21 and 22. Figure 15A shows the foldchange of the genes (AKT, FoXO1, IRa, IRb, and GPR151) in the livers of the different female groups (SD, HFD, SDMeth, HFDMeth, 5 mg/L, and 20 mg/L). AKT expression showed a significant increase (*P* < 0.05) in the HFD and HFDMeth groups (7.19 (± 0.56) and 6.13 (± 1.11), respectively) compared to the SD group (1 (± 1.68)). The SD and SDMeth (0.59 (± 0.17)) groups showed no significant difference (*P* > 0.05). Similarly, the treated group with 5 mg/L (2.79 (± 1.24)) and 20 mg/L (2.71 (± 1.09)) of DL-limonene showed no significant difference (*P* > 0.05) compared to the SD group; however, it showed a significant decrease to the HFD and HFDm groups.

Among the male liver groups, the housekeeping gene GAPDH had a Ct value mean ranging between 23 and 25. Figure 15B shows the fold change of the genes (AKT, FoXO1, IRa, IRb, and GPR151) in the different groups of the male liver (SD, HFD, SDm, HFDm, 5 mg/L, and 20 mg/L). AKT showed a significant increase (*P* < 0.05) in the HFD and HFDMeth groups (3.46 (± 0.08)) compared to the SD group (1 (± 0.86)). The treated groups with 5 mg/L (1.49 (± 0.01)) and 20 mg/L (2.43 (± 0.3)) of DL-limonene showed no significant difference (*P* > 0.05) compared to the SD group; however, the group treated with 5 mg/L was the only group that showed a significant decrease comparing to the HFD group. The group treated with 20 mg/L showed significant differences to SDMeth but not HFD and HFDm groups.

Among the female brain groups, the housekeeping gene GAPDH had a Ct value mean ranging between 20 and 22. Figure 15C shows the fold change of the genes (AKT, FoXO1, Ira, IRb, and GPR151) in the different groups of the female brain (SD, HFD, SDm, HFDm, 5 mg/L, and 20 mg/L). The fold change shows no significant difference (*P* > 0.05) between the groups in all tested genes.

Among the male brain groups, the housekeeping gene GAPDH had a Ct value mean ranging between 23 and 25. Figure 15D shows the fold change of the genes (AKT, FoXO1, IRa, IRb, and GPR151) in the brains of fish in the different groups (SD, HFD, SDm, HFDm, 5 mg/L, and 20 mg/L). The expression of the AKT gene showed a significant decrease (*P* < 0.05) in the groups HFD (0.41 (± 0.18)), SDm (0.1 (± 0.04)), HFDm (0.38 (± 0.06)), 5 mg/L (0.2 (± 0.01)) and 20 mg/L (0.08 (± 0.02)) compared to SD group (1(± 0.06)). The group treated with 20 mg/L showed a significant decrease in AKT expression (*P* < 0.05) compared to the HFD group. However, the rest of the genes (FoXO1, IRa, IRb, and GPR151) showed no significant difference (*P* > 0.05) compared to the SD group.

## Discussion

It has been reported that 5 mg of artemia per day matches the metabolic energy requirements of an adult zebrafish. Thus, raising the usual calorie demands is necessary to raise zebrafish weight and, as a result, BMI. In this study, we report an effective the feeding procedure to develop a zebrafish diabetes model in 4 weeks, rather than 8 weeks as was reported in the previous protocol^16^.

DL-limonene was found to successfully decrease the BMI and fasting blood glucose levels of male and female diabesity zebrafish. This result is consistent with the findings of Bacanli et al. on the preventive impact of limonene in diabetic rats^4^. In other studies, limonene has reduced the proliferation of white and brown adipocytes, decreased serum triglyceride and fasting blood glucose levels, and inhibited liver lipid accumulations^17^.

The metabolites of HFD zebrafish were identified based on the chemical shift data of the standard compounds available in the Chenomx library. Caprate, 2-hydroxybutyrate, 3-methylglutarate, 3-hydroxybutyrate, galactarate, allantoin, *N*-acetylcysteine, isovalerate, kynurenine, and 2-hydroxyisocaproate were among the lipophilic metabolites that increased. Among aqueous metabolites, glucose and acetoacetate were the only ones to rise, while the rest of the hydrophilic metabolites declined including isoleucine, creatine, tyramine, taurine, citrate, valine, leucine, alanine, lactate, *O*-phosphocholine, acetate, galactose, choline, *O*-acetylcarnitine, glycylproline, histidine, methionine, histamine, 3-hydroxyisovalerate, phenylalanine, and citrate.

The detected metabolites in male and female diabesity zebrafish might be classified according to the pathway in which they participate:

### Glucose/energy metabolism pathway

Glucose levels in this study were significantly higher in both male and female diabesity zebrafish groups, as shown by glucometer measurement and metabolomic fingerprinting. Glucose in the cell is transformed to glucose-6-phosphate as the initial stage in the degradation of glucose through glycolysis, a process that is critical for energy production. The second step of glycolysis is the conversion of glucose-6-phosphate to fructose-6-phosphate by glucose-6-phosphate isomerase, or ribose-5-phosphate by glucose-6-phosphate dehydrogenase to enter the pentose pathway. it was reported that high glucose in the case of diabetes leads to inhibition of glucose-6-phosphate dehydrogenase^18^. In addition, as the first stage in the hexosamine biosynthesis process, glucosamine-6-phosphate is produced from fructose 6-phosphate and glutamine. This process generates UDP-*N*-acetylglucosamine, which is subsequently utilized to synthesize glycosaminoglycans, proteoglycans, and glycolipids. Numerous investigations have shown that fructose concentrations rise in the serum and urine in diabetes^19^. It has been reported that sex matters when it comes to sucrose, which is elevated in obese male animal models but not in females^20^. Fucose levels in diabetic individuals have been also observed to be higher^21^. However, the decrease in galactose levels in the diabetes female zebrafish group might be attributed to an increase in excretion in urine, which is common in diabetes^22^.

Glycolysis is completed when glucose-6-phosphate is converted to pyruvate and then to acetyl CoA, which enters the tricarboxylic acid cycle (TCA). Notably, *O*-acetylcarnitine, which is increased in male obese zebrafish, increases the transport of acetyl-CoA into the matrix of mitochondria during fatty acid oxidation. In this research, the analysis of diabesity female zebrafish revealed a decrease in citric acid levels as compared to non-diabesity groups. Diabetes causes an increase in citric acid in the blood and urine as a result of diabetes and nephropathy^23^. ^1^H NMR metabolomics investigations in diabetic mice also reveal an increase in lactate as a result of anaerobic glucose metabolism^24^. Lactate might be decreased in case of high activity of lactate dehydrogenase enzyme which converts lactate to pyruvate^25^.

### Amino acids metabolism

Due to a change in energy metabolism, increased levels of amino acids and their metabolites in the blood as a result of protein catabolism have been linked to insulin resistance and type 2 diabetes in epidemiological studies^26^. Glycylproline is a dipeptide consisting of L-proline having a glycyl residue attached to its alpha-amino group. Glycylproline levels are reduced in diabesity female zebrafish in this research, as well as in other animal models previously reported (Makowski et al., 2014; Mora-Ortiz et al., 2019). In addition, It was discovered that type 2 diabetes patients had an elevated level of 2-hydroxybutyrate^27^. Additionally, 2-hydroxybutyrate has a positive correlation with haemoglobin A1C (HbA1C), which increases throughout diabetes and is often measured to assist in the diagnosis and monitoring of patients with diabetes^28^. Besides, 2-hydroxyisovalerate and 2-hydroxyisocaproate are metabolites associated with Branched-chain amino acids (BCAAs) matabolism and it was reported to be increased in type 2 diabetes animal models and humans^29^. BCAAs (isoleucine, leucine, and valine), alanine, methionine, and phenylalanine are catabolized to supply carbon skeletons to the TCA, which is used to generate energy^30^. Accordingly, both male and female diabetic zebrafish have lower levels of BCAAs, alanine, and phenylalanine. It was reported that plasma histamine and histidine concentrations are elevated in patients and animal models with type 2 diabetes mellitus and obesity^31^. In this research, the concentrations of histamine and histidine differ between male and female diabetic zebrafish. The tyramine byproduct of tyrosine metabolism has been shown to reduce diabesity in female zebrafish. Patel et al.^32^ discovered that patients with metabolic syndrome had lower tyramine levels than healthy individuals.

Tryptophan is metabolized through the kynurenine pathway which depends on the expression of indoleamine-2, 3-dioxygenase, and tryptophan-2,3-dioxygenase. Kynurenine is further processed to produce several major metabolites: 3-hydroxykynurenine and quinolinic acid are synthesized by kynurenine-3-monoxygenase, whereas kynurenic acid is synthesized in the presence of kynurenine aminotransferase. Type 2 diabetes patients have greater kynurenine serum levels and lower indoleamine tryptophan-2,3-dioxygenase activity^33^. 3-Hydroxy-3-methylglutarate is an intermediate metabolite in the amino acid leucine degradation process^34^. Along with earlier investigations, it has been observed that 3-hydroxy-3-methylglutarate levels rise in diabetes animal models^35^. Besides, *N*-acetylglutamate is required for carbamyl phosphate synthetase I activation, the first enzyme in the urea cycle, and fluctuations in its concentration affect the rate of urea production^36^. The increased *N*-acetylglutamate concentration might be due to the high activity of *N*-acetylglutamate synthase, which results in increased hepatic urea cycle synthesis^37^. 5-Aminopentanoate is one of the lysine catabolites, and it is a by-product of arginine and proline metabolism^38,39^. As found in this report, 5-aminopentanoate was also increased in previous diabetic investigations^40^. *N*-acetylcysteine is reported to be increased in type 2 diabetes animal models^41^. The metabolites creatinine, betaine, and creatine were observed to be increased in this study in diabesity male zebrafish. Creatine is transported from the liver to tissues such as skeletal muscle and the brain, where it is phosphorylated to form phosphocreatine which is used as a short-term energy source. According to our findings, reatine levels drop in diabetic patients, particularly those with diabetic retinopathy^42^. Creatine is also linked to choline, which is transformed into betaine, creatine, and finally creatinine in the case of type 2 diabetes^43^.

### Fatty acid oxidation pathways

In the case of caloric restriction, the liver breaks down fatty acids in a mechanism known as ketogenesis to create energy, resulting in an increase in ketone bodies in the blood. The accumulation of ketone bodies (acetone, acetoacetate, and 3-hydroxybutyrate) causes diabetic ketoacidosis, which is one of the diabetes complications^44^. Branched-chain fatty acids (isovalerate and 3-methylglutarate) are saturated fatty acids having one or more methyl branches on the carbon chain. Little is known about the involvement of this type of fatty acid in diabetes; nonetheless, it has been linked to obesity and diabetes^45^. Elevated free fatty acids and intracellular lipids limit GLUT-4 translocation, which inhibits insulin-stimulated muscle glucose transport^46^. Sebacate is a medium-chain fatty acid that has been identified as a biomarker of metabolic syndrome^47^. Caprate and 2-oxohexanoic acid were identified as a biomarker for hereditary type 2 diabetes^48^. Mahendran et al. 2013 find out a correlation between the increased level of free fatty acids and glycerol in diabetes patients^49^. The increasing concentration of glycerol in the bloodstream is a result of the body's greater reliance on lipids as a source of energy. On the other hand, glycerol may be used in both glycolysis and gluconeogenesis^50^.

### Uric acid and bile acids metabolites

Bile acids increase the formation of glucagon-like peptide 1 in the distal small intestine and colon, which stimulates insulin release and, as a result, is implicated in carbohydrate and fat metabolism^51^. Cholate, like chenodeoxycholic acid, is the main bile acid produced in the liver from cholesterol. In addition, the uric acid cycle is a cyclic adaption of the purine catabolism process. It is widely established that diabetes may increase urea cycle metabolites such as allantoin^52^.

The ^1^H NMR fingerprint analysis confirms that DL-limonene may lower carbohydrate levels, including glucose, in both male and female zebrafish. Limonene was also able to change the fatty acid metabolites, suggesting that limonene regulates the fatty acid pathways. Limonene has been shown in vitro to have a role in the regulation of specific genes, including hormone-sensitive lipase, perilipin, and AMP-activated protein kinase, which are related to lipolysis and lipid metabolism^53^.

Several types of animal models are commonly used in biomedical research, including rodents (e.g., mice and rats) and larger animals (e.g., pigs or non-human primates). Several endogenous metabolites that have been investigated as potential diagnostic biomarkers for type 2 diabetes in different animal models include glucose metabolites (glucose, glycated hemoglobin (HbA1c), and markers of glycolysis or gluconeogenesis), lipid metabolites (triglycerides, free fatty acids, and cholesterol derivatives), and amino acid metabolites (branched-chain amino acids (BCAAs), aromatic amino acids, and urea cycle intermediates)^54,55^.

Insulin produced from the pancreas binds to a particular isoform receptor (IRa and IRb) in the liver, adipose tissue, and muscles, triggering a massive phosphorylation cascade inside the targeted cells. The insulin receptor activation activates an adaptor protein known as the insulin-receptor substrate. The insulin-receptor substrate activates the lipid kinase phosphatidylinositol-4,5-bisphosphate 3-kinase (PI3K), which phosphorylates phosphatidylinositol-4,5-bisphosphate (PIP2) to generate phosphatidylinositol-3,4,5-trisphosphate (PIP3). PIP3 then interacts with AKT in the cytoplasm, promoting glucose transporter gene activation. The Akt activation requires other cytoplasmic protein interactions, which are 3-phosphoinositide-dependent protein kinase-1 (PDK-1) and the mammalian target of rapamycin complex 2 (mTORC2). The converted PIP3 from PIP2 facilitates the phosphorylation of AKT in the threonine-308 by PDK-1. Partial AKT activation is a suffusion to stimulate protein synthesis^56^. However, suppression of Forkhead O Family 1 (FoxO1), which stimulates glucose synthesis, requires mTORC2 phosphorylation of AKT at serine-473. FoxO1 phosphorylation promotes its translocation from the nuclease to the cytosol, where it degrades and therefore inhibits the transcription of glucose-production enzymes^57^. Thus, IRa, IRb, AKT, and FoxO1 have been investigated in this study in the liver and brain of tested zebrafish. The findings revealed no statistically significant differences in the expression of IRa, IRb, and FoxO1 across the groups in the liver and brain. On the other hand, AKT gene expression was raised in the HFD groups but reduced after DL-limonene treatment.

It is well known that insulin-dependent AKT phosphorylates and inactivates glycogen synthase kinase (GSK)-3, enhancing glycogen synthase activity and accelerating glycogen synthesis. However, it is also confirmed that AKT could be upregulated independently of insulin and promote glycogen synthesis^58^. AKT expression, for instance, increased dramatically in diabetic patients after exercise training^59^. Besides, Diabetes induces oxidative stress in the liver, which is xidativezed by an increase in the number of reactive oxygen species (ROS) in tissue and a significant reduction in antioxidant defences^60^. It was observed that there was an increase in the expression of AKT in the liver as a result of the emergence of oxidative stress^61^. In addition, it was confirmed that limonene protects the liver from xidativee stress due to its antioxidant and anti-inflammatory capabilities^62^. Collectively, our findings imply that DL-limonene might mitigate the oxidative stress caused by diabetes-induced HFD via increasing AKT expression. However, this hypothesis needs more experimentation to be proven. Besides, AKT stimulates hexokinase to convert glucose to glucose-6-phosphate in intracellular compartments^56^. Our findings indicated that both AKT and glucose-6-phosphate levels increased.

## Conclusion

We have established a type 2 diabetes model by feeding male and female zebrafish an HFD. Although both males and females had high blood glucose levels and BMI, the endogenous metabolites differed by gender. The model was utilized to assess the activity of the natural compound DL-limonene. Bioinformatics analysis of SD and HFD of zebrafish demonstrated that these biomarkers were involved in glucose/energy metabolism, amino acid metabolism, lipid metabolism, bile acids, and uric acid metabolism. Based on the findings, future studies should consider the gender of zebrafish while screening for diabetes drugs. The study decreases the duration of diabetes induction from 8 to 4 weeks only, which will save the researchers time in future research. DL-limonene could be a candidate therapeutic agent since it: (1) decreases both BMI and blood glucose; (2) reverses the changes in metabolites due to diabesity; (3) participates in protein and lipid metabolism; (4) may help regulate genes and enhance gene expression that is impaired by diabesity. However, further investigations are needed to confirm these effects.

## Data Availability

The datasets used and/or analysed during the current study available from the corresponding author on reasonable request.
